# The future of artificial intelligence in medicine: Medical-legal considerations for health leaders

**DOI:** 10.1177/08404704221082069

**Published:** 2022-03-31

**Authors:** Sunam Jassar, Scott J. Adams, Amy Zarzeczny, Brent E. Burbridge

**Affiliations:** 1 12371 University of Saskatchewan, Saskatoon, Saskatchewan, Canada.; 2 6846 University of Saskatchewan, Saskatoon, Saskatchewan, Canada.; 3 University of Regina, Regina, Saskatchewan, Canada.

## Abstract

Artificial Intelligence (AI) is becoming increasingly common in healthcare and has potential to improve the efficiency and quality of healthcare services. As the utility of AI expands, medical-legal questions arise regarding the possible legal implications of incorporating AI into clinical practice. Particularly, the unique black box nature of AI brings distinct challenges. There is limited guidance addressing liability when AI is used in clinical practice, and traditional legal principles present limitations when applied to novel uses of AI. Comprehensive solutions to address the challenges of AI have not been well established in North America. As AI continues to evolve in healthcare, appropriate guidance from professional regulatory bodies may help the medical field realize AI’s utility and encourage its safe use. As the options for AI in medicine evolve, physicians and health leaders would be prudent to consider the evolving medical-legal context regarding use of AI in clinical practices and facilities.

## Introduction

Artificial Intelligence (AI) is becoming increasingly common in healthcare, with applications ranging from screening and triage to clinical risk prediction and diagnosis.^1,2^ As a clinical tool, AI has the potential to improve diagnostic accuracy and the efficiency of health services.^3^ While AI can incorporate a spectrum of diverse tasks, broadly, it refers to computer-based technology systems that can simulate human intelligence in order to perform tasks or actions.^3^ Further evolution of AI in medicine provides a unique opportunity to enhance patient care but creates potential new risks by redefining the nature of physician involvement.^4^ As AI continues to gain increased autonomy and demonstrates the ability to perform accurately in the absence of physician input, medical-legal questions may arise regarding the possible legal implications for physicians and health systems incorporating AI into clinical practice.

This analysis article will (1) describe the current legal landscape of the clinical use of AI in Canada, (2) discuss how legal principles may be applied across a spectrum of uses for AI and the limitations of applying these traditional principles to emerging and novel uses of medical AI, (3) discuss examples of emerging international guidance surrounding the use of AI, and (4) propose that guidance from professional bodies can help promote the appropriate and safe use of medical AI. These considerations will be relevant for health leaders and managers as they consider the implications of expanding uses of medical AI in their jurisdictions and healthcare facilities.

### What is the current legal landscape of medical AI in Canada?

**Figure 1.**
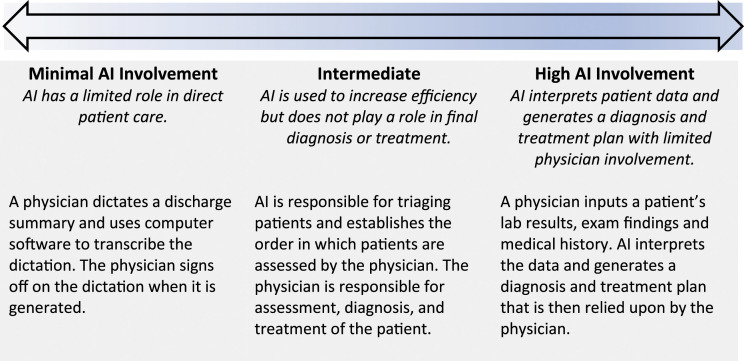
Spectrum of AI use in medicine.

There is a wide spectrum of tasks that can be performed by AI, with varying degrees of agency (Figure 1). Common applications of AI in medicine range from voice recognition software for clinical and administrative documentation to computer-aided detection of abnormalities in medical imaging.^3^ These tasks currently require input from physicians as the main drivers of decision-making. Legal uncertainty, however, arises when AI is employed to perform novel tasks with greater independence from physicians, or when physicians rely on information from an AI algorithm which may be unverifiable (ie, a so-called “black box”). Health Canada considers AI as a medical device pursuant to the *Food and Drugs Act* but evolving forms and uses of AI may test the limits of current regulations.^5^ There is also limited legal precedent addressing potential liability if patient harm results from the use of AI.^6^

With the rise of AI in healthcare, task forces have been established to discuss these developing issues and potential future strategies. In the 2021 federal budget, the Canadian government announced a 10-year renewal of the Pan-Canadian Artificial Intelligence Strategy.^7^ As part of this strategy, the government aims to take advantage of the growth opportunities in AI while working toward advancing the development of AI standards.^7,8^ The Royal College of Physicians and Surgeons of Canada formed a task force which considered, among other things, the ethical and legal aspects of AI and other emerging technologies.^9^ The Royal College’s report recognized that with increasing advancements in AI, current legal regimes lack clarity.^9^ In particular, liability and accountability become an issue if harm arises from AI-driven medical decisions.^9^

The Canadian Medical Association (CMA) has created specific policies on medicine and technology following the rise of technology in healthcare.^10^ One of the guiding principles of the CMA’s policy on recommending mobile health applications to patients is that mobile applications are meant to be used as tools to enhance patient care and not to replace the physician-patient relationship.^11^ Current policies do not specifically address the use of AI in medicine or the possibility of AI-driven medical decisions. In the face of limited legal precedent or policies guiding the clinical use of AI, uncertainty about responsibility, accountability, and liability of physicians and health systems risks serving as a chill on this promising field.

### What legal principles apply to AI in medicine?

In Canada, physicians may be liable for negligence when a patient suffers harm or loss as a result of the physician’s breach of the standard of care. In determining the standard of care, courts compare the physician’s actions against the standard of a reasonable and prudent physician in similar circumstances, considering the specialty, level of training, setting, and resources available.^12^ Past court decisions analyzing liability in negligence for harms suffered following use of other medical technologies can provide guidance on how courts might address medical liability associated with the use of AI.

Under its current use, AI operates largely as a clinical tool used by physicians.^13,14^ However, certain uses of medical AI that involve increased agency may present novel legal challenges, such as the use of AI to diagnose lung cancer.^15^ Clinicians relying on imaging alone cannot establish with certainty whether a particular lung nodule is benign or malignant.^16^ AI, using large data sets of validated pathologic imaging, can assess lung nodules on CT and generate a risk score which informs physicians of the probability of malignancy.^15^ AI’s accuracy is contingent on the reference data set that the algorithm uses to learn to identify malignant lung nodule characteristics.^15^ The data set can vary in its representation of certain population characteristics and this may impact the accuracy of its assessment. As the complexity of AI algorithms become more difficult to comprehend, they are referred to as “black boxes” to reflect their potential lack of transparency.^17^ The black box nature of AI processes may result in an inability to assess what characteristics were relied upon by the algorithm to generate an assessment. If a physician relies on AI’s findings, and it is incorrect or inaccurate, there may be implications for the patient in the form of either being subject to unnecessary treatment, unnecessary follow-up procedures or an undiagnosed malignancy. The unique black box nature of AI, and the potential for its increased autonomy, may differentiate it from other commonly used clinical decision tools, and may raise new legal questions, including the relevant standard of care when using AI in practice. The different legal principles that may apply to the clinical use of AI are outlined in Table 1.

**Table 1.** Applying traditional legal principles to medical AI.

Legal Principles	Definition	Application
**Negligence**	The defendant has a duty of care to the plaintiff, the defendant failed to meet the standard of care, and that breach caused harm to the patient.^18^	• The components of the standard of care required of physicians using AI are unclear, particularly where AI is a “black box” and/or operates with a level of autonomy not common to other medical technologies. • It is unclear what information needs to be shared with patients to obtain properly informed consent when AI is used in diagnosis and treatment recommendations.
**Strict Liability**	The defendant is held legally responsible for the harm caused in the absence of a finding of fault or negligence.^19^	• Provides greater access to compensation for harms suffered and reduces the burden of litigation on plaintiffs. • This heightened standard of liability could limit the use of AI and hamper medical AI innovation.
**Vicarious Liability**	Liability is imposed on the principal (eg, employer) for the actions of its agent (eg, employee).^20^	• Potential for liability to be imposed on the physician and/or the medical institution using AI in practice.
**Product Liability**	Imposes a continuous duty on the manufacturer to warn consumers or the intermediary of the dangers arising from the use of the product.^21^	• The black box nature of AI may make it difficult to identify the risks associated with its use. • The source of the defect or cause of the risk or error may be difficult to trace to its origin.

### Is there international guidance on the liability of AI in medicine?

International guidance varies with Europe and the United States, for example, taking different approaches to addressing the legal challenges AI may pose to healthcare. The European Union (EU) has been at the forefront of medical AI innovation and has explicitly recognized the challenges AI presents for existing liability regimes.^22^ To harmonize liability principles and provide legal certainty, the European Commission has proposed one of the first legal frameworks specific to AI, the *Artificial Intelligence Act*.^22^ Through this framework, the European Commission aims to promote the safe use of AI in high impact sectors, such as healthcare, while also strengthening technological innovation.^22^

The European Parliament made recommendations to the Commission on a civil liability regime for AI to address accountability and compensation principles.^23^ The proposed liability regime purports that a complete revision of existing liability principles is currently unnecessary and, notably, does not allow AI the ability to act independently or assume responsibility in law.^23^ The liability regime uses existing legal principles and adopts a risk-based approach distinguishing high risk AI systems from low risk AI systems.^23^ To address inherent risk and increased autonomous nature, high risk AI systems are subject to strict liability while other AI systems fall under a negligence based liability scheme.^23^ Medical devices that fall within the EU’s existing medical device regulations are specifically classified as high risk and are therefore subject to strict liability - a liability standard that can establish liability in the absence of fault or negligence.^19,22,23^ Under this regime, operators, either persons exercising control over the operation or the features of the technology, can be liable for injuries arising from AI.^23^ This liability approach for medical AI addresses the concerns of accountability and compensation but may inadvertently impede innovation of AI in medicine. The framework attempts to evade the black box problem by extending liability onto individuals involved in the creation, maintenance, or control of AI systems.^23^ While the success of this proposed framework remains to be seen, it may ultimately become a blueprint for other countries to follow.

The United States, like Canada, does not have a single established legal framework governing AI and there is limited legal precedent regarding liability and medical AI. As in Canada, determining the relevant standard of care will be central to questions of liability but may also be a challenge in this fast-moving field. From a regulatory perspective, the U.S. Food and Drug Administration (FDA) has recognized the challenges AI can pose and seeks to promote the safe use of AI in healthcare through an action plan to maintain oversight of AI as a medical device.^24^ To promote a patient-centred approach, the FDA aims to increase transparency by asking manufacturers to describe the functioning of their AI devices to better understand benefits and risks.^24^ The FDA also aims to overcome bias that can occur when AI algorithms are trained using a specific population or historical datasets.^24^

### What are possible future directions for medical AI?

The future of medical AI is promising and shows that AI has the potential to improve healthcare delivery.^14^ While AI currently has a relatively limited role in direct patient care, its evolving role in complex clinical decision making is foreseeable.^14^ As the technology develops and different uses expand, novel legal questions may arise with respect to liability for harms suffered. Past precedent will offer some guidance, but there is the potential that uncertainty and fear of liability may impede the development and uptake of these technologies.

Depending on the European Union’s success with its *Artificial Intelligence Act,* its approach may serve as a useful model for promoting uniformity in governance of AI technologies. Increasing pressure from task forces calling for legal clarity, and Canada’s recent renewal of the Pan-Canadian AI Strategy, in combination with evolving AI technology, could mean a domestic framework is on the horizon.^8,9^ One of the potential advantages of such efforts is that they may promote public confidence in AI by providing oversight while simultaneously encouraging innovation. In the meantime, however, professional regulatory bodies could play an important role by providing guidance to their members and establishing expectations regarding relevant standards of care. As the field develops, it will be important for the medical community and regulators to monitor the explainability of AI technologies, as well as their agency, and consider how those factors impact the use of AI in different healthcare contexts.

In the absence of a concrete legal framework, guidance from a pan-Canadian multistakeholder group—including, for example, the CMA, Royal College of Physicians and Surgeons of Canada, College of Family Physicians of Canada, Medical Council of Canada, Federation of Medical Regulatory Authorities of Canada, provincial medical regulatory authorities, and Canadian Nurses Association, among others—could be a valuable step in increasing uniformity, increasing physician and healthcare provider confidence, and promoting safe use of AI in clinical practice. Guiding principles relating to the scope of AI, communicating the use of AI with patients to obtain informed consent, and assessing the use and application of AI is a pivotal step in establishing a standard practice. Proactive leadership from professional bodies may help foster public confidence in the safety and utility of medical AI, and fuel future innovation in this promising field.

## Conclusion

Continued advances of AI in healthcare may offer significant benefit to healthcare providers and patients alike. However, potential benefits of AI also come with possible risk and uncertainty. Unlike other clinical tools, AI’s black box nature and potential for increased agency may present distinct challenges in the legal realm. Well established and comprehensive solutions to address the challenges AI presents have yet to be developed nationally and internationally. As this new technological era in healthcare evolves, timely guidance from professional bodies may help realize AI’s utility in transforming healthcare delivery and to encourage its appropriate use in medical settings.

## References

[1] Levin S Toerper M Hamrock E . Machine-learning-based electronic triage more accurately differentiates patients with respect to clinical outcomes compared with the emergency severity index. Ann Emerg Med. 2018;71(5):565-574. 28888332 10.1016/j.annemergmed.2017.08.005

[2] Gulshan Peng L Coram M , et al. Development and validation of a deep learning algorithm for detection of diabetic retinopathy in retinal fundus photographs. JAMA. 2016;316(22):2402-2410. 27898976 10.1001/jama.2016.17216

[3] Chen M Decary M . Artificial intelligence in healthcare: an essential guide for health leaders. Healthc Management Forum. 2020;33(1):10-18. 10.1177/0840470419873123 31550922

[4] Allain J . From jeopardy! ZZo jaundice: the medical liability implications of Dr. Watson and other aritificial intelligence systems. LA Law Rev. 2013;73(7):1049-1079.

[5] Canadian Institutes of Health Research. Introduction of Artificial Intelligence and Machine Learning in Medical Devices . 2019 May. Available: https://cihr-irsc.gc.ca/e/51459.html. (accessed 2021 Sept 10).

[6] Jaremko JL Azar M Bromwich R , . Canadian association of radiologists white paper on ethical and legal issues related to artificial intelligence in radiology. Can Assoc Radiol J. 2019;70(2):107-118. 30962048 10.1016/j.carj.2019.03.001

[7] Budget . Building an innovation economy of the future. Department of Finance Canada; modified 2021 Apr. 19. Available: 2021. https://www.canada.ca/en/department-finance/news/2021/04/budget-2021-building-an-innovation-economy-of-the-future.html. (accessed 2021 Aug. 6).

[8] Stragety Pan-Canadian AI . Canadian institute for advanced research. 2021 Aug. 8. Available: https://cifar.ca/ai/ (accessed 2021 Aug. 8)

[9] Reznick R Harris K Horsley T Sheikh M . Task force report on artificial intelligence and emerging digital technologies. 2020 Feb. Available: https://www.royalcollege.ca/rcsite/health-policy/initiatives/ai-task-force-e. (accessed 2021 July 13).

[10] Canadian Medical Association . CMA policy base. 2021 Jun. 28. Available: https://policybase.cma.ca/en. https://policybase.cma.ca/en. (accessed 2021 Jun. 28).

[11] Canadian Medical Association. Guiding principles for physicians recommending mobile health applications to patients . Available: 2015 May 30. https://policybase.cma.ca/en/viewer?file=%2fdocuments%2fPolicyPDF%2fPD15-13.pdf#search=mobile%20apps&phrase=false. https://policybase.cma.ca/en/viewer?file=%2fdocuments%2fPolicyPDF%2fPD15-13.pdf#search=mobile%20apps&phrase=false. (accessed 2021 Jun. 28).

[12] Wilson v. Swanson , 1956 CANLII 1 (SCC). [1956] SCR 804. Available: https://canlii.ca/t/1nlkb

[13] Price WN Gerke S Cohen IG Cohen IG . Potential liability for physicians using artificial intelligence. JAMA. 2019;322(18):1765-66. 31584609 10.1001/jama.2019.15064

[14] Verma AA Murray J Greiner R , et al. Implementing machine learning in medicine. Canadian Medical Association Journal. 2021;193(34):E1351-E1357. 35213323 10.1503/cmaj.202434 PMC8432320

[15] Mindshare Medical . Mindshare medical announces health Canada clearance for nationwide use of revealai-lung product. 2018. Available: https://www.newswire.ca/news-releases/mindshare-medical-announces-health-canada-clearance-for-nationwide-use-of-revealai-lung-product-689882931.html. (accessed 2021 Aug. 8).

[16] MacMahon H Naidich DP Goo JM , et al. Guidelines for management of incidental pulmonary nodules detected on CT images: from the fleischner Society 2017. Radiology. 2017;284(1):228-243. 28240562 10.1148/radiol.2017161659

[17] Bathaee Y . The artificial intelligence black box and the failure of intent and causation. Harv J Law Technol. 2018;31(2):890-938.

[18] Durbin E . Torts – nature of tort law and liability. west law Canada. 2021. Available: https://www.westlawnextcanada.com/academic/ced/torts. (accessed 2021 Aug 8).

[19] Rylands v. Fletcher , 1868 UKHL 1, [1868] LR 3 HL 330.

[20] Bazley v. Curry , 1999 CanLII 692 (SCC), [1999] 2 SCR 534. Available: https://canlii.ca/t/1fqlw.

[21] Hollis v. Dow Corning Corp , 1995 CanLII 55 (SCC), [1995] 4 SCR 634. Available: https://canllii.ca/t/1frdr.

[22] Proposal for a Regulation of The European Parliament and of The Council Laying Down Harmonised Rules on Artificial Intelligence (Artificial Intelligence Act) And Amending Certain Union Legislative Acts . Brussels: European Commission; 2021. Available: https://eur-lex.europa.eu/resource.html?uri=cellar:e0649735-a372-11eb-9585-01aa75ed71a1.0001.02/DOC_1&format=PDF. (accessed 2021 June 5).

[23] Civil liability regime for artificial intelligence . European Parliament resolution of 2020 with recommendations to the Commission on a civil liability regime for artificial intelligence. Brussels: European Parliament; 2020. https://www.europarl.europa.eu/doceo/document/TA-9-2020-0276_EN.pdf. (accessed 2021 June 5).

[24] Artificial intelligence/machine learning (AI/ML) – based software as a medical device (SaMD) action plan. Food & Drug Administration . 2021. Available: https://www.fda.gov/media/145022/download. (accessed 2021 Aug 8).

